# Deciphering Alloy Composition in Superconducting Single-Layer FeSe_1–*x*_S*_x_* on SrTiO_3_(001) Substrates by Machine Learning of STM/S Data

**DOI:** 10.1021/acsami.2c23324

**Published:** 2023-05-01

**Authors:** Qiang Zou, Basu Dev Oli, Huimin Zhang, Joseph Benigno, Xin Li, Lian Li

**Affiliations:** † Department of Physics and Astronomy, West Virginia University, Morgantown, West Virginia 26506, United States; ‡ Lane Department of Computer Science and Electrical Engineering, West Virginia University, Morgantown, West Virginia 26506, United States

**Keywords:** alloy composition, single-layer iron chalcogenides, MBE, STM/S, machine learning

## Abstract

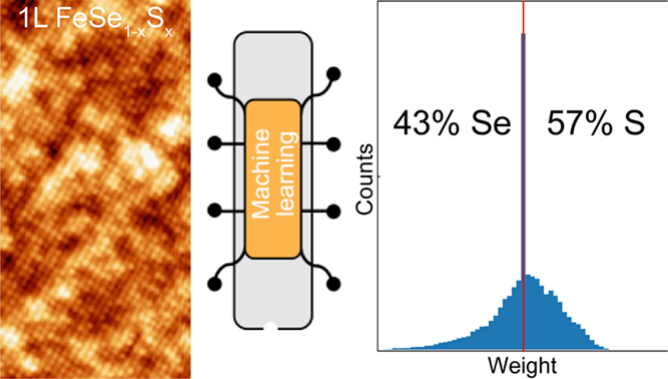

Scanning tunneling microscopy (STM) is a powerful technique for imaging atomic structure and inferring information on local elemental composition, chemical bonding, and electronic excitations. However, a plain visual analysis of STM images can be challenging for such determination in multicomponent alloys, particularly beyond the diluted limit due to chemical disorder and electronic inhomogeneity. One viable solution is to use machine learning to analyze STM data and identify hidden patterns and correlations. Here, we apply this approach to determine the Se/S concentration in superconducting single-layer FeSe_1–*x*_S*_x_* alloys epitaxially grown on SrTiO_3_(001) substrates via molecular beam epitaxy. First, the K-means clustering method is applied to identify defect-related d*I*/d*V* tunneling spectra taken by current imaging tunneling spectroscopy. Then, the Se/S ratio is calculated by analyzing the remaining spectra based on the singular value decomposition method. Such analysis provides an efficient and reliable determination of alloy composition and further reveals the correlations of nanoscale chemical inhomogeneity to superconductivity in single-layer iron chalcogenide films.

## Introduction

Chemical doping is often exploited to tune material properties.^1−7^ For example, in semiconductors, aliovalent substitution leads to p- and n-type doping, the key elements of devices such as diodes and transistors that enable applications in modern electronics such as information processing, sensing, energy harvesting, and medical devices.^8−13^ Though a critical component, such substitutions can also introduce chemical and electronic inhomogeneities at the nanoscale, which can significantly impact the carrier mobility and performance of semiconducting devices. These inhomogeneities can be particularly detrimental with shrinking device dimensions, including the recent emergence of two-dimensional semiconducting monolayered materials.^14−17^ On the other hand, for quantum materials, these heterogeneities are usually correlated and give rise to quantum phenomena such as entanglement and topological effects, magnetism, and superconductivity.^3−6,18^ For instance, vacancy defects in wide bandgap semiconductors, such as diamond and silicon carbide, can result in tightly bound and robust spin states even at room temperature. Such quantum states are promising candidates for qubits that can be entangled with their neighbors and addressed by both optical and microwave techniques.^18^ In addition, most cuprate superconductors exhibit a significant amount of chemical disorder since the isovalent or aliovalent substitutions that are substantially required to achieve superconductivity frequently lead to lattice site vacancies and/or interstitials.^3−6^ Such structural knowledge is often obtained using scattering techniques, including X-ray and neutron scattering, where recent advances in X-ray scattering have enabled insight into chemical bonds with picometer resolution.^8,19,20^ Nonetheless, most such studies are inherently limited to macroscopically averaged properties. Alternatively, atomic scale imaging and spectroscopy by scanning tunneling microscopy (STM) and transmission electron microscopy (TEM) have been key to deciphering chemical disorder and electronic heterogeneity in quantum materials.^21−23^ Regardless, a visual analysis of STM and TEM images can be challenging for multicomponent alloys, particularly beyond the diluted limit due to chemical disorders and electronic inhomogeneities. One way to address this challenge is to use computational methods/statistical analysis (e.g., fast Fourier transform) to analyze the data obtained from imaging experiments to identify patterns and correlations that may not be immediately apparent through visual inspection alone.^24−26^ A recent approach is to apply machine learning (ML) algorithms to analyze STM/TEM images, where they can be trained to recognize patterns and correlations in the data.^27^ Such an approach has been applied to identify hidden electronic orders in STM imaging of quantum matter and to determine surface chemical bonding and adatom interactions.^28−30^

**Figure 1:**
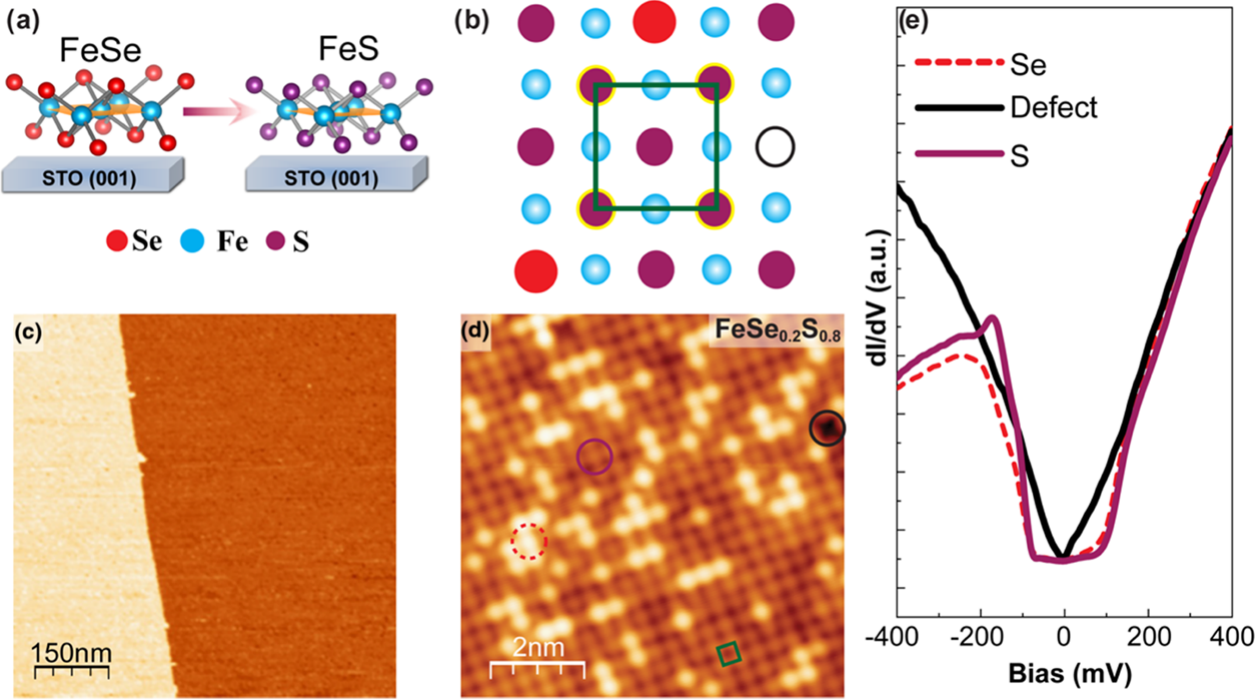
STM image and tunneling spectra of single-layer FeSe_0.2_S_0.8_ on SrTiO_3_(001) substrates. (a) Ball-and-stick model of isovalent substitution of S into single-layer FeSe on STO substrates. (b) Top-view schematic of single-layer FeSe_1–*x*_S*_x_* alloy (open circle represents a Se/S vacancy defect). (c) Large-scale and (d) atomic-resolution STM image of single-layer FeSe_0.2_S_0.8_/STO. Imaging conditions: 3 V, 100 pA for panel (c) and 20 mV, 500 pA for panel (d). The green square in panel (d) marks the unit cell, and the black solid circle marks a Se/S vacancy. (e) Tunneling spectra taken at the three locations marked in panel (d). Set point: *V*_Bias_ = 400 meV and *I*_T_ = 500 pA.

Here, we determine S and Se concentrations based on machine learning of STM imaging of single-layer (SL) FeSe_1–*x*_S*_x_*. Iron chalcogenides exhibit a layered structure consisting of a plane of iron atoms with chalcogen (S, Se, Te) residing alternately above and below the plane, as sketched in Figure 1a,b. This structure enables the epitaxial growth of iron chalcogenide thin films on most substrates.^31−33^ For SL FeSe films grown on SrTiO_3_(001) (STO) by molecular beam epitaxy (MBE), the superconducting transition temperature has been enhanced by nearly 1 order of magnitude, the highest superconducting transition temperature in iron-based materials to date.^34^ Nonetheless, the mechanism for Cooper pairing in such a system is still under debate, with several theories proposed including plain s-wave driven by electron–phonon coupling and d-wave driven by antiferromagnetic spin fluctuations.^35−41^ To address this issue, we have shown that the superconductivity in SL FeSe/STO can be tuned by S doping, similar to earlier studies.^42−44^ In the case of S substitution, chemical pressure caused by the size difference between Se and S shortens the height of Fe–Se, thus tuning its paramagnetic ground state and superconductivity.^45^ In this approach, the local chemical composition is a critical parameter that can be obtained by visual inspection of atomic-resolution STM images. However, chemical disorders and electronic inhomogeneities make this determination challenging beyond the diluted limit.

To determine the Se/S ratio for films with intermediate alloy compositions, ML methods are employed to analyze current imaging tunneling spectroscopy (CITS) in two steps. First, we separate the defect-related features based on the analysis of d*I*/d*V* tunneling spectra using the supervised K-means clustering method. Next, the remaining defect-free tunneling spectra are sorted into Se and S groups using the unsupervised singular value decomposition (SVD) method, which reduces CITS maps to a dataset with significantly fewer values and captures a large fraction of their variabilities (detailed in the Supporting Information). This analysis results in the Se/S ratio and also reveals the correlations between nanoscale chemical inhomogeneity and superconductivity. Our findings demonstrate an effective and reliable approach for determining alloy composition in single-layer superconducting iron chalcogenide films.

## Results and Discussion

Single-layer FeSe_1–*x*_S*_x_* were epitaxially grown on STO substrates, followed by 450 °C annealing for 1 h, where the S concentration *x* was controlled by adjusting the evaporating temperature of the S source from 300 to 400 °C during growth (The Methods section and Supporting Information Table S1). The surface morphology of SL FeSe_1–*x*_S*_x_*/STO films was examined by STM measurements, where it is conformal to the step-terrace topography of the STO substrate (Figure 1c). At the diluted limit of up to ∼20% of Se (or S), their positions can be identified by the conventional visual inspection of atomic-resolution STM images and d*I*/d*V* tunneling spectra. For example, because of their larger size, the Se atoms exhibit higher contrast in STM images as marked by the red dashed circle in Figure 1d. The difference in Se and S can also be found in the d*I*/d*V* tunneling spectra as shown in Figure 1e. While the spectra acquired at Se and S atoms are both U-shaped near the Fermi level, the valence band edge shifts from −210 meV for Se to −180 meV for S. By counting the numbers of Se and S atoms in the STM image, we determined that the Se concentration is 20% for this film (see Figure S1, Supporting Information for details and additional example). Note that a common type of defect observed is the Se/S vacancy, as marked by the black circle in Figure 1d. Such a defect also caused a change of d*I*/d*V* tunneling spectra to be V-shaped for the whole energy range between −400 and 400 meV, which is dramatically different from those of Se and S sites.

**Figure 2:**
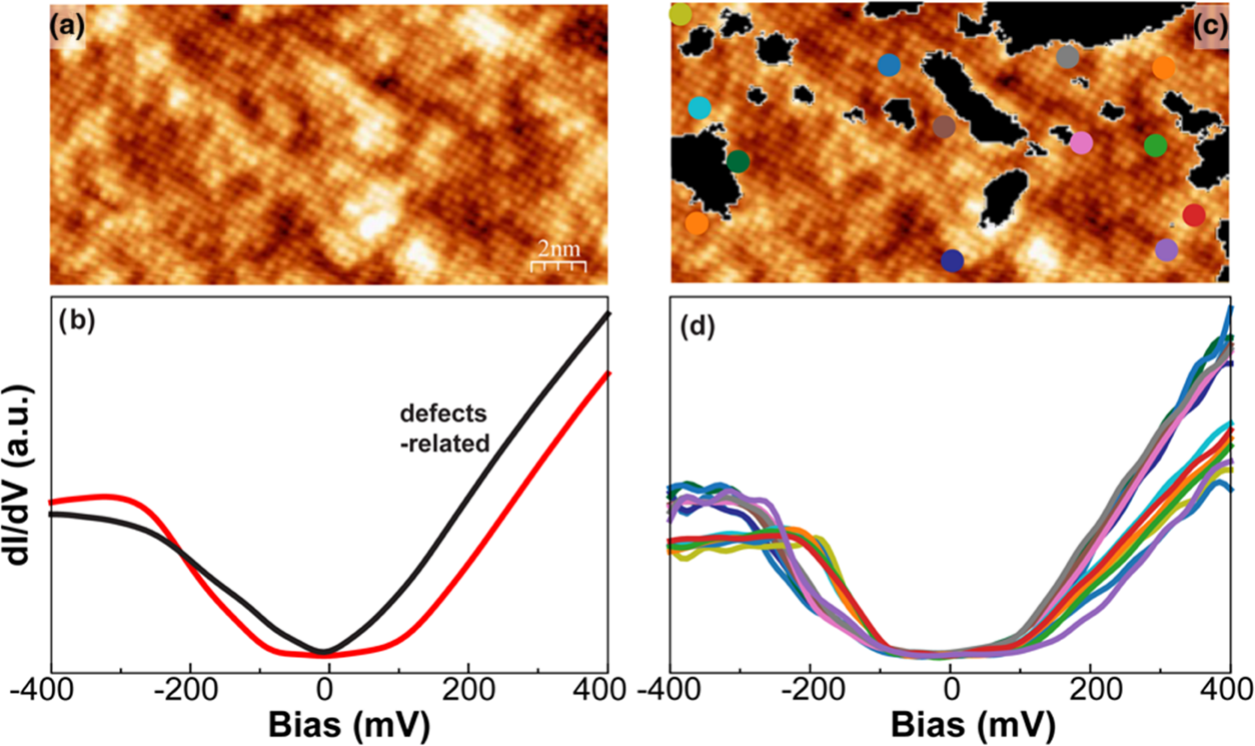
K-means clustering analysis of defects in single-layer FeSe_0.43_S_0.57_. (a) Atomic-resolution image of single-layer FeSe_0.43_S_0.57_. Imaging conditions: 100 mV and 100 pA. (b) Average tunneling spectra of the two clusters from K-means clustering of current imaging tunneling spectroscopy in panel (a). (c) Clusters map from K-means plotted over the atomic-resolution image. (d) Tunneling spectra at the spots marked in panel (c). Set point: *V*_Bias_ = 400 meV and *I*_T_ = 500 pA.

The above method of visually inspecting atomically resolved STM images works well for determining the Se/S concentrations in SL FeSe_1–*x*_S*_x_*/STO films in the diluted limit (see Figure S1, Supporting Information for another example). However, it becomes increasingly challenging for Se/S concentrations between 0.3 and 0.7 because of the higher density of defects and electronic inhomogeneity present in the STM images. This is illustrated in Figure 2a for the intermediate concentration case FeSe_0.43_S_0.57_, where the Se sites are hard to distinguish from those of S because of various sites with multiple high and low contrasts, which is also bias-dependent (see Figure S2, Supporting Information for bias-dependent STM images). To determine the Se/S ratio in such a complex system, we apply ML to analyze CITS maps in two steps: (1) identify the defect-related tunneling spectra in the CITS map by the K-means clustering method, a supervised vector quantization data mining approach that groups a large dataset into components with principal characteristics and (2) sort out the remaining (defect-free) CITS map into Se and S groups by the singular value decomposition (SVD) method.^46−48^ (Note that the single-step approach, including K-means, SVD, and principal component analysis, cannot capture the composition variations due to the chemical, electronic, and structural inhomogeneities at the nanoscale, as shown in Supporting Information Figures S3–S5).

In the first step, for the film shown in Figure 2a, tunneling spectra in the CITS map are cataloged into two groups, as shown in Figure 2b. The first group displays the characteristic V-shaped tunneling spectrum of defects and the second group, the U-shaped (details of the K-means clustering method are provided in Supporting Information Figure S3). Then, the spatial distribution of those two groups (V- and U-shaped) of tunneling spectra is overlaid on the atomic-resolution STM image, which reveals that the V-shaped spectra are located primarily at the very dark and the most bright locations. This analysis indicates that the defect regions are associated with V-shaped d*I*/d*V* tunneling spectra and thus are excluded from the calculations of the Se/S ratio. Next, d*I*/d*V* spectra are selected randomly in the rest of the CITS map as shown in Figure 2d. Interestingly, those tunneling spectra merge into two groups, characteristic of those for Se and S for films in the diluted limit (cf., FeSe_0.2_S_0.8_ in Figure 1e).

**Figure 3:**
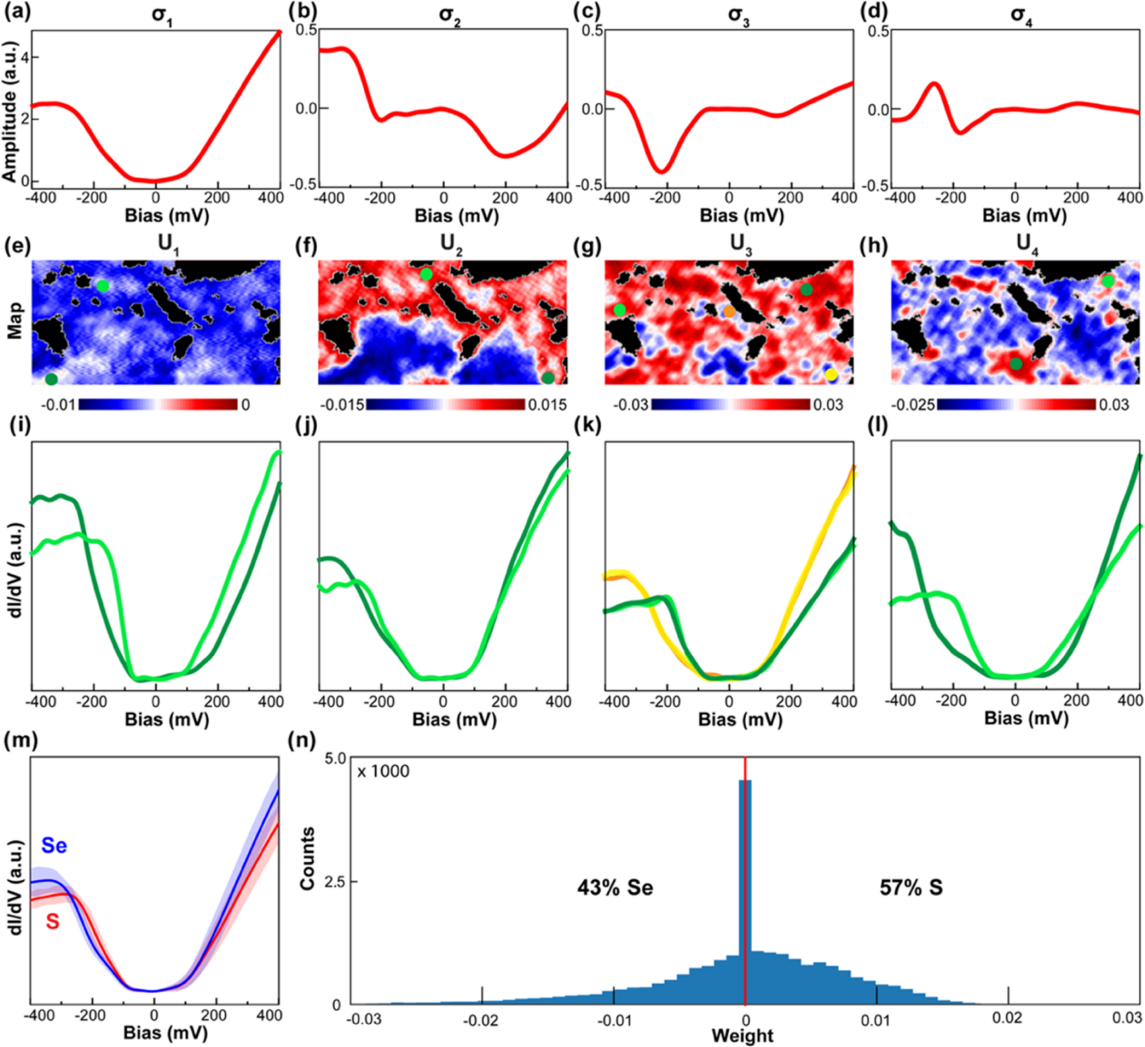
Determination of the Se (S) concentration in single-layer FeSe_0.43_S_0.57_. Panels (a, e), (b, f), (c, g), and (d, h) are the σ_*i*=1,2,3,4_ and corresponding spatial distribution map U_*i*=1,2,3,4_, obtained by singular value decomposition, respectively. (i–l) Tunneling spectra were taken at the spots indicated in panels (e–h). (m) Average tunneling spectra with standard deviations at the red and blue locations of map U_3_. (n) Statistics of spatial distribution map U_3_.

To quantitatively determine the Se and S concentrations, we utilize the SVD method to decompose the remaining tunneling spectra. The SVD is one of the most important matrix factorizations in computation, providing a numerically stable matrix decomposition and the optimal low-rank approximation for high-dimensional data.^48^ Briefly, the optimal rank-r approximation to the matrix A is described by
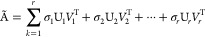
where *k* ∈(1,2,···*r*) is the significant rank index, the columns of U ({U*_i_*}) are called the left singular vectors, the columns of *V*({*V_i_*}) are called the right singular vectors, and the {σ_*i*_^2^} (σ_1_ > σ_2_··· > σ*_r_*) are the eigenvalues of AA^T^ (more details of the SVD method is provided in the Supporting Information). For the film FeSe_0.43_S_0.57_, the four leading square root of eigenvalues (σ_*i*=1,2,3,4_) and their corresponding spatial distribution maps ({U_*i*=1,2,3,4_}) are shown in Figure 3a–h, respectively (additional square root of eigenvalues and their spatial distribution maps are presented in Supporting Information Figure S6).

Close examination of the distribution maps reveals that the spatial distribution map U_3_ successfully represents the spatial distribution of Se and S. Although the first leading square root of eigenvalue σ_1_ (Figure 3a) is U-shaped, the corresponding spatial distribution map U_1_ mixes the Se with S groups. Two spots are randomly selected from the same white color scheme in U_1_ as indicated by the bright and dark green points in Figure 3e, and then their corresponding tunneling spectra from the raw CITS dataset are plotted in Figure 3i. The two tunneling spectra are clearly different, indicating that the U_1_ does not distinguish Se from S. Similar failure is also found for other spatial distribution maps, such as U_2_, and U_4_. As shown in Figure 3f and h, the two tunneling spectra in the red color scheme in each U_2,4_ are distinctive. In contrast, for U_3_ (Figure 3g), the tunneling spectra at dark and bright green points in the red color scheme are mostly identical (Figure 3k). Moreover, the tunneling spectra at yellow and orange spots in the blue color scheme also overlapped. Those observations confirm that the U_3_ successfully represents the spatial distribution of Se and S. It also indicates that the σ_3_ captures the difference in tunneling spectra between Se and S in this sample.

We further confirm the successful grouping of U_3_ by checking spatially dependent tunneling spectra presented in Supporting Information Figure S7. The evolution of tunneling spectra from Se (U-shaped) to defect-related (V-shaped) to S (U-shaped) region is consistent with the U_3_ map. Therefore, we concluded that the left singular vector U_3_ displays the spatial distribution of Se in the blue color scheme and S in the red color scheme. The average tunneling spectra with standard deviations from Se and S atoms based on U_3_ are presented in Figure 3m. Furthermore, based on the histogram of the spatial distribution U_3_ (Figure 3n), we determine that the concentration of S (Se) is 57% (43%). This ML method was also applied successfully to determine the alloy composition of other SL FeSe_1–*x*_S*_x_*/STO (results for FeSe_0.51_S_0.49_ are presented in Supporting Information Figure S8).

**Figure 4:**
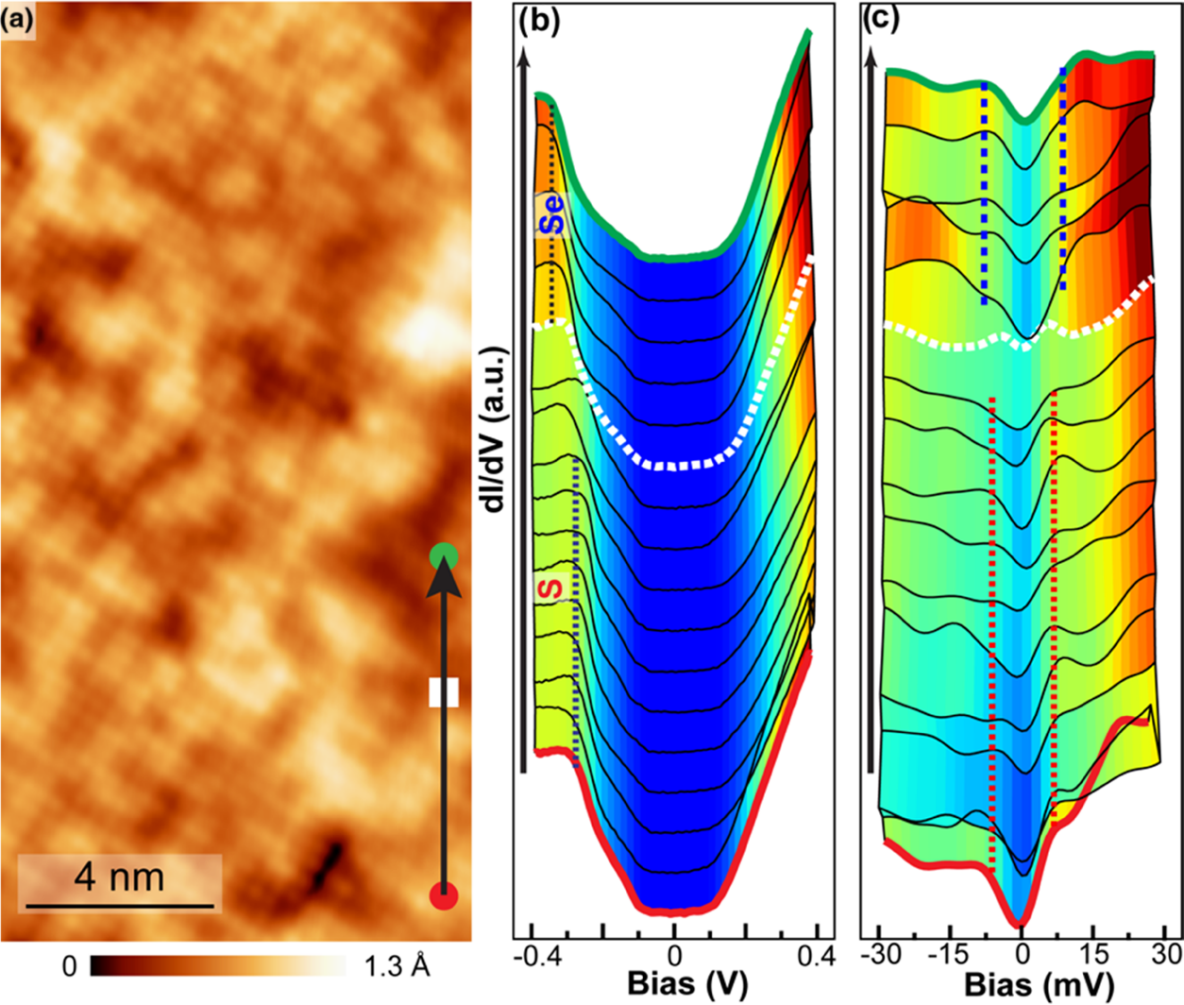
Line STS in single-layer FeSe_0.43_S_0.57_. (a) Atomic-resolution image. (b, c) Line STS along the black arrow in panel (a) in a large bias range and around the Fermi level, respectively. Set point: *V*_Bias_ = 400 meV, *I*_T_ = 500 pA, and *V*_Bias_ = 30 meV, *I*_T_ = 500 pA. *T* = 4.3 K. The vertical red and blue lines in panel (c) serve as a guide to the eye for the superconducting coherence peaks.

The above analysis shows that the electronic structure of SL FeSe_1–*x*_S*_x_*/STO films exhibits high spatial inhomogeneity. Importantly, this inhomogeneity impacts the superconductivity locally, as indicated by the variation of the amplitudes and positions of superconducting coherence peaks in tunneling spectra shown in Figure 4c. Based on d*I*/d*V* tunneling spectra taken along the black line in Figure 4a, the valence band edge varies from −266 meV at S atoms to −327 meV at Se atoms, consistent with the results of Figure 3. Near the Fermi level, one pair of superconducting coherence peaks are clearly observed, while the gap values are correlated with the Se/S concentration. For the S-rich regions, the gap obtained from the energy positions of superconducting coherence peaks is smaller with an average value of Δ_1_ = 6.5 ± 1 meV, while the Se-rich regions exhibit a slightly larger value of Δ_2_ = 8.0 ± 1 meV. Hence, the superconductivity of iron chalcogenide is tuned by the chemical pressure by the S substitution.

## Conclusions

High-quality single-layer FeSe_1–*x*_S*_x_* films are epitaxially grown on SrTiO_3_(001) by MBE and investigated using *in situ* low-temperature scanning tunneling microscopy/spectroscopy. For films at the diluted limit, the Se/S ratio is determined by the traditional visual inspection of atomic-resolution STM images and d*I*/d*V* tunneling spectra. However, for films with intermediate alloy compositions where such an approach is challenging, we combine unsupervised with supervised machine learning methods on the spatially dependent tunneling spectra to determine the chemical compositions. This ML-based approach provides an effective and reliable method to determine alloy concentrations and further reveal strong correlations between nanoscale chemical inhomogeneity and superconductivity in single-layer iron chalcogenide films.

## Methods

### Molecular Beam Epitaxy Growth

Single-layer FeSe_1–*x*_S films were grown on Nb-doped (0.5% wt) SrTiO_3_ (001) substrates using molecular beam epitaxy. The SrTiO_3_ (001) substrates were annealed at 1050 °C for 1 h in an ultrahigh vacuum (UHV) MBE chamber (base pressure ∼1 × 10^–9^ Torr). After that treatment, the SrTiO_3_ (001) surfaces show atomically flat terraces and sharp step edges, confirmed by in situ STM. The growth of single-layer FeSe_1–*x*_S films was carried out on the annealed SrTiO_3_ (001) substrates. Fe was supplied using an electron beam source, Se/S from two separate Knudsen cells. The growth rate was about 0.5 monolayer/min, which was controlled by the Fe flux. The evaporating temperature of the Se source was generally kept at 106 °C during growth, and the S concentration was tuned by adjusting the evaporating temperature of the S source (ferrous sulfide crystal) from 300 to 400 °C (more details in Supporting Information Table S1). The flux ratio of Fe/Se(S) is estimated to be 1:10–20. During the growth, the substrates were at 250 °C and as-grown samples were further annealed at about 450 °C for 1 h to achieve superconductivity. For each concentration, multiple samples were grown, and their Fermi surface and band structures were monitored by *in situ* angle-resolved photoemission spectroscopy to ensure reproducibility.

### Scanning Tunneling Microscopy/Spectroscopy

The scanning tunneling microscopy and spectroscopy (STM/S) measurements were carried out in a Unisoku ultrahigh vacuum low-temperature STM SPM-1300 system with a base temperature of 4.3 K. A polycrystalline PtIr tip was used and tested on Ag/Si(111) films before the STM/S measurements. Tunneling spectra were acquired using a standard lock-in technique with a small bias modulation V_mod_ (2% of the setting point bias) at 732 Hz.

### K-Means

The K-means method groups a large dataset into components with quintessential characteristics. The K-means clustering analysis uses a supervised learning algorithm to find similar groups in the data, where the number of groups is represented by the variable K. We used the same K-means codes as in ref (46).

### Singular Value Decomposition (SVD)

SVD is one of the most widely used multivariate statistical techniques for matrix decomposition. The purpose of singular value decomposition is to reduce a dataset containing a large number of values to a dataset containing significantly fewer values, which can still capture a large fraction of the variability present in the original data. We used the numpy.linalg.svd module to perform SVD analysis.^49^

## Supporting Information Available

SVD method; evaporation temperatures for Se and S sources (Table S1); determination of S concentrations in single-layer alloys by visual inspection at the diluted limit (Figure S1); bias-dependent STM images of single-layer FeSe_0.43_S_0.57_ (Figure S2); K-means analysis of CITS spectra of single-layer FeSe_0.43_S_0.57_ (Figure S3); PCA of the CITS of single-layer FeSe_0.43_S_0.57_ (Figure S4); SVD analysis of the CITS of single-layer FeSe_0.43_S_0.57_ (Figure S5); SVD analysis of the defect-free CITS of single-layer FeSe_0.43_S_0.57_ (Figure S6); line d*I*/d*V* spectra crossing regions of different Se/S ratios in single-layer FeSe_0.43_S_0.57_ (Figure S7); and determination of the Se (S) concentration in single-layer FeSe_0.51_S_0.49_ (Figure S8) (PDF)
