# Supplementary material for: Deciphering Alloy Composition in Superconducting Single-Layer FeSe1–xSx on SrTiO3(001) Substrates by Machine Learning of STM/S Data
Source: ACS Appl Mater Interfaces. 2023 May 1;15(18):22644–50. doi: 10.1021/acsami.2c23324 (PMC10176460; doi:10.1021/acsami.2c23324)
Supplement: Supplementary file 1 — am2c23324_si_001.pdf [file am2c23324_si_001.pdf]

SUPPORTING INFORMATION

Deciphering alloy composition in superconducting single-layer $FeSe_{1-x}S_x$ on $SrTiO_3$(001) substrates by machine learning of STM/S data

Qiang Zou[†], Basu Dev Oli[†], Huimin Zhang[†], Joseph Benigno[†], Xin Li[‡], Lian Li[†,*]

[†]Department of Physics and Astronomy, West Virginia University, WV 26506, USA

[‡]Lane Department of Computer Science and Electrical Engineering, West Virginia University, WV 26506, USA

*To whom correspondence may be addressed. E-mail: lian.li@mail.wvu.edu

SVD method

For the SVD method, a two-dimensional dataset is decomposed into $A = U \Sigma V^T$, where $U \in C^{(a\times b)\times m}$ and $V \in C^{n\times c}$ are unitary matrices 1 with orthonormal columns, and $\Sigma \in R^{m\times n}$ is a matrix with real, nonnegative entries on the diagonal and zeros off the diagonal. The columns of U are called the left singular vectors and the columns of V are called the right singular vectors. Furthermore, we can prove that $AA^T = U\Sigma^2U^T$, where $\{U_i\}$ are the eigenvectors of AA^T with eigenvalues $\{\sigma_i^2\}$.

Generally, the most useful feature of the SVD method is that it provides an optimal low-rank approximation of a matrix A. In fact, the SVD provides a hierarchy of low-rank approximations since a rank-r approximation is obtained by keeping the leading r singular values and vectors and discarding the rest. In the least squares sense, the optimal rank-r approximation to the matrix A is given by the rank-r SVD truncation:

$$\frac{\text{argmin}}{\tilde{A},\ s.t. rank(\tilde{A}) = r} ||A - \tilde{A}||_F = \tilde{U}\tilde{\Sigma}\tilde{V} \quad (1)$$

,where $\tilde{U}$ and $\tilde{V}$ denote the first r leading columns of U and V, and $\tilde{\Sigma}$ contains the leading $r \times r$ sub-block of Σ, $\|\cdot\|_F$ the Frobenius norm. Because Σ is diagonal, the rank-r SVD approximation is given by the sum of r distinct rank-1 matrices:

$$\tilde{A} = \sum_{k=1}^{r} \sigma^1 U_1 V_1^T + \sigma^2 U_2 V_2^T + \cdots + \sigma^r U_r V_r^T. \quad (2)$$

Therefore, high-dimensional data A can be approximately well described by a few dominant patterns given by the columns of $\tilde{U}$ and $\tilde{V}$.

Supplementary Table S1| Evaporate temperatures for Se and S sources.

Sample	T_{se} (°C)	T_S (°C)
FeSe	106	0
$FeSe_{0.43}S_{0.57}$	106	330
$FeSe_{0.51}S_{0.49}$	106	340
$FeSe_{0.2}S_{0.8}$	106	380
$FeSe_{0.06}S_{0.94}$	98	400
FeS	0	360

Supplementary Figures

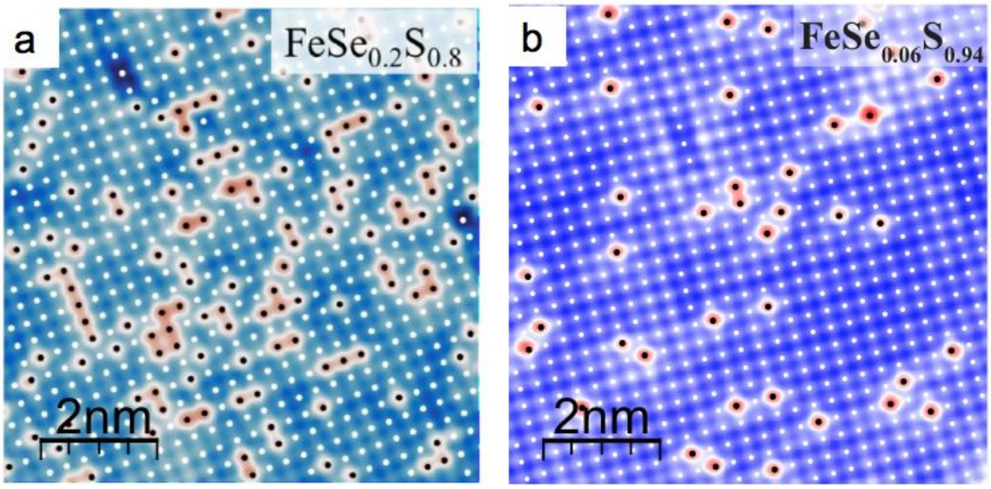

Fig. S1 | Determination of S concentrations in single-layer alloys by visual inspection at the diluted limit. Atomic resolution STM images of single-layer $FeSe_{0.2}S_{0.8}$ (a) and $FeSe_{0.06}S_{0.94}$ (b). The bright spots are Se atoms, as marked in black dots. The Se and S concentration are determined by counting the numbers of S and Se atoms in each atomic resolution image. Imaging conditions: 20 mV, 500 pA.

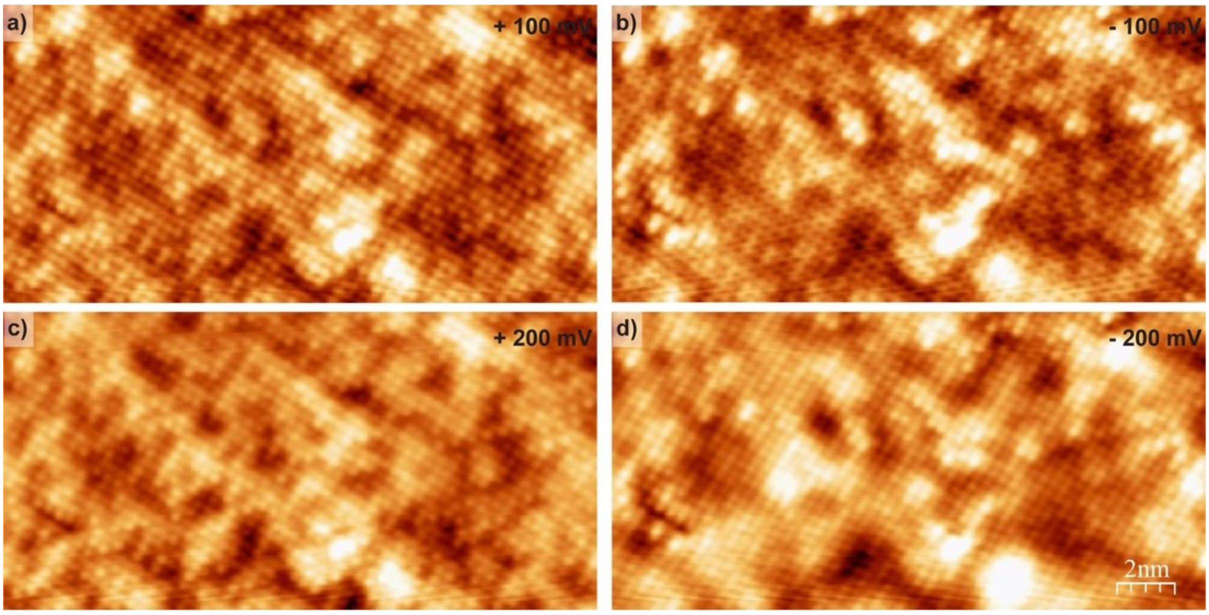

Fig. S2 | Bias-dependent STM images of single-layer $FeSe_{0.43}S_{0.57}$. (a)-(d) Images are acquired at the same location with marked bias voltages. The tunneling current (I) is set to 100 pA for all cases.

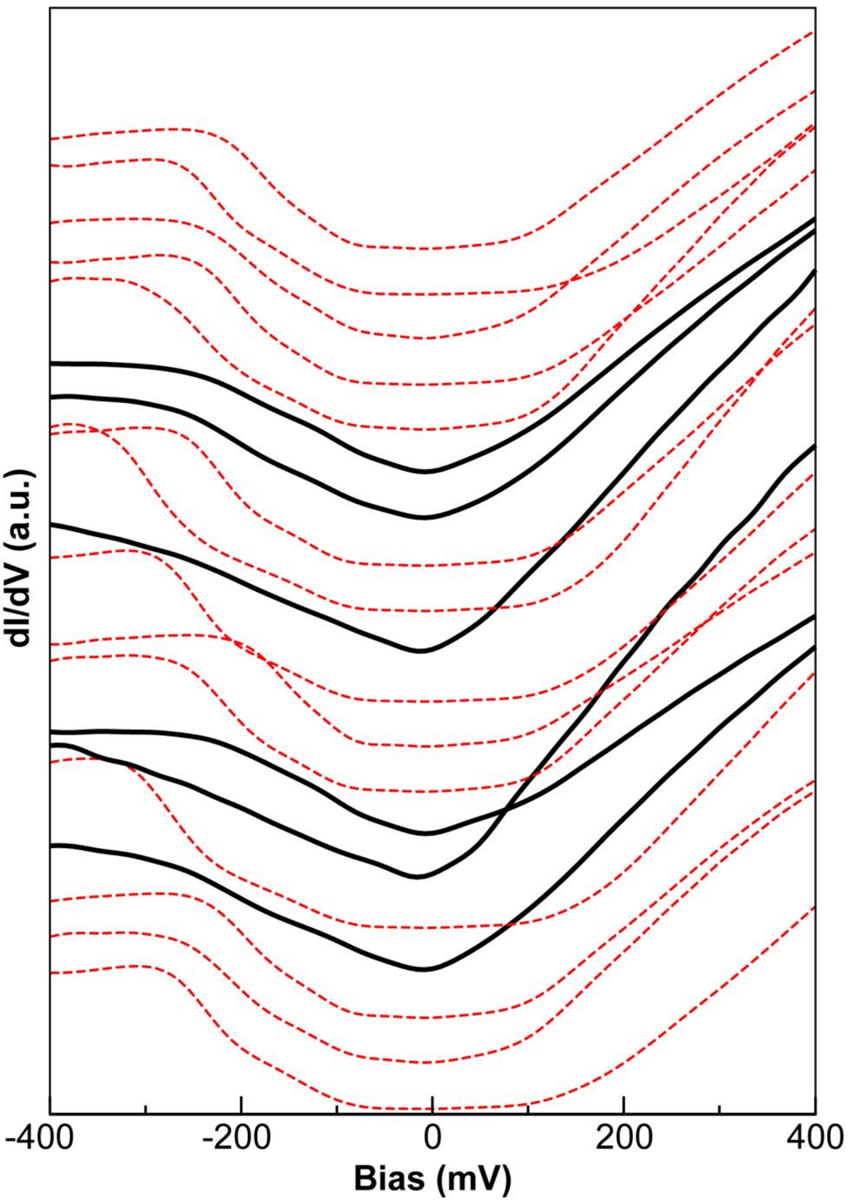

Fig. S3 | K-means analysis of CITS spectra of single-layer $FeSe_{0.43}S_{0.57}$. The twenty K-means principal responses of the 21,632 spatially dependent tunneling spectra of single layer $FeSe_{0.43}S_{0.57}$, which can be grouped into two types of line shapes: (red dash) U-shaped and (black solid) V-shaped.

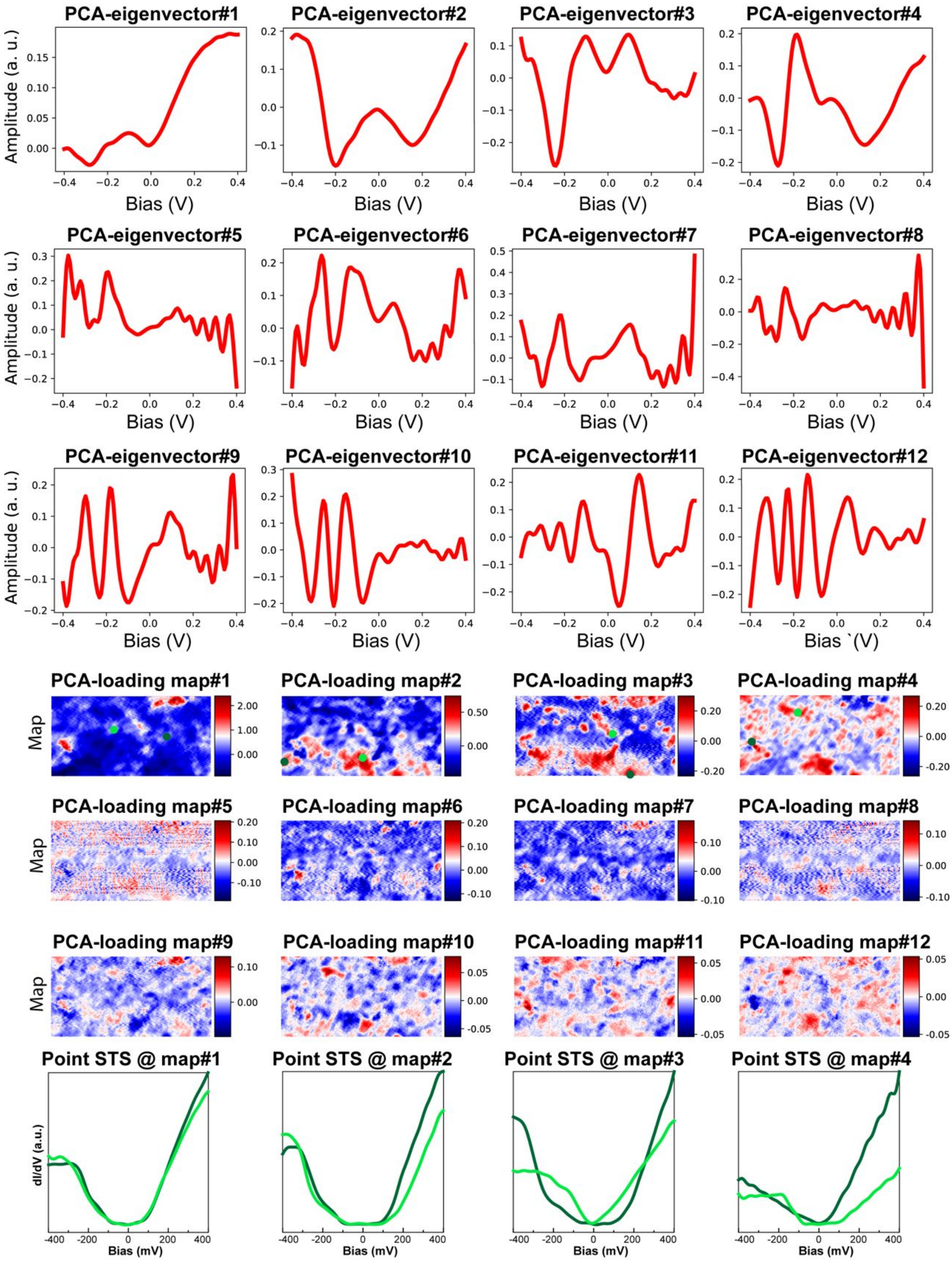

Fig. S4 | Principal component analysis (PCA) analysis of the CITS of single-layer $FeSe_{0.43}S_{0.57}$. The twelve leading of eigenvectors and their corresponding loading map are obtained by PCA analysis. STS at the same color scheme were compared in the loading map 1, 2, 3, and 4.

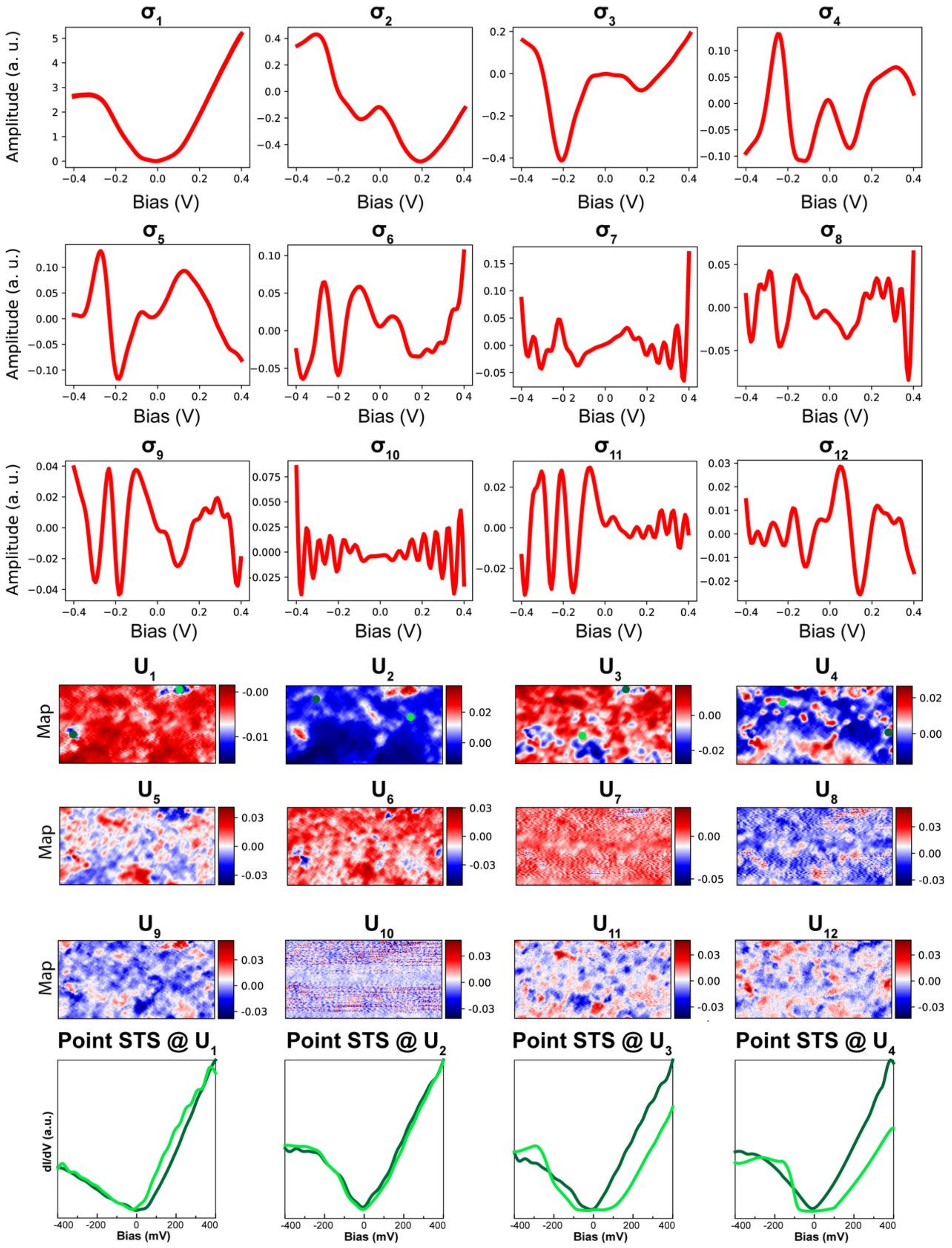

Fig. S5 | Singular value decomposition analysis of the CITS of single-layer $FeSe_{0.43}S_{0.57}$. The twelve leading of $\sigma_{i=1, 2, \ldots, 12}$ and their corresponding spatial distribution map $U_{i=1, 2, \ldots, 12}$ are obtained by singular value decomposition. STS at the same color scheme were compared in the $U_{i = 1, 2, 3, \text{and } 4}$.

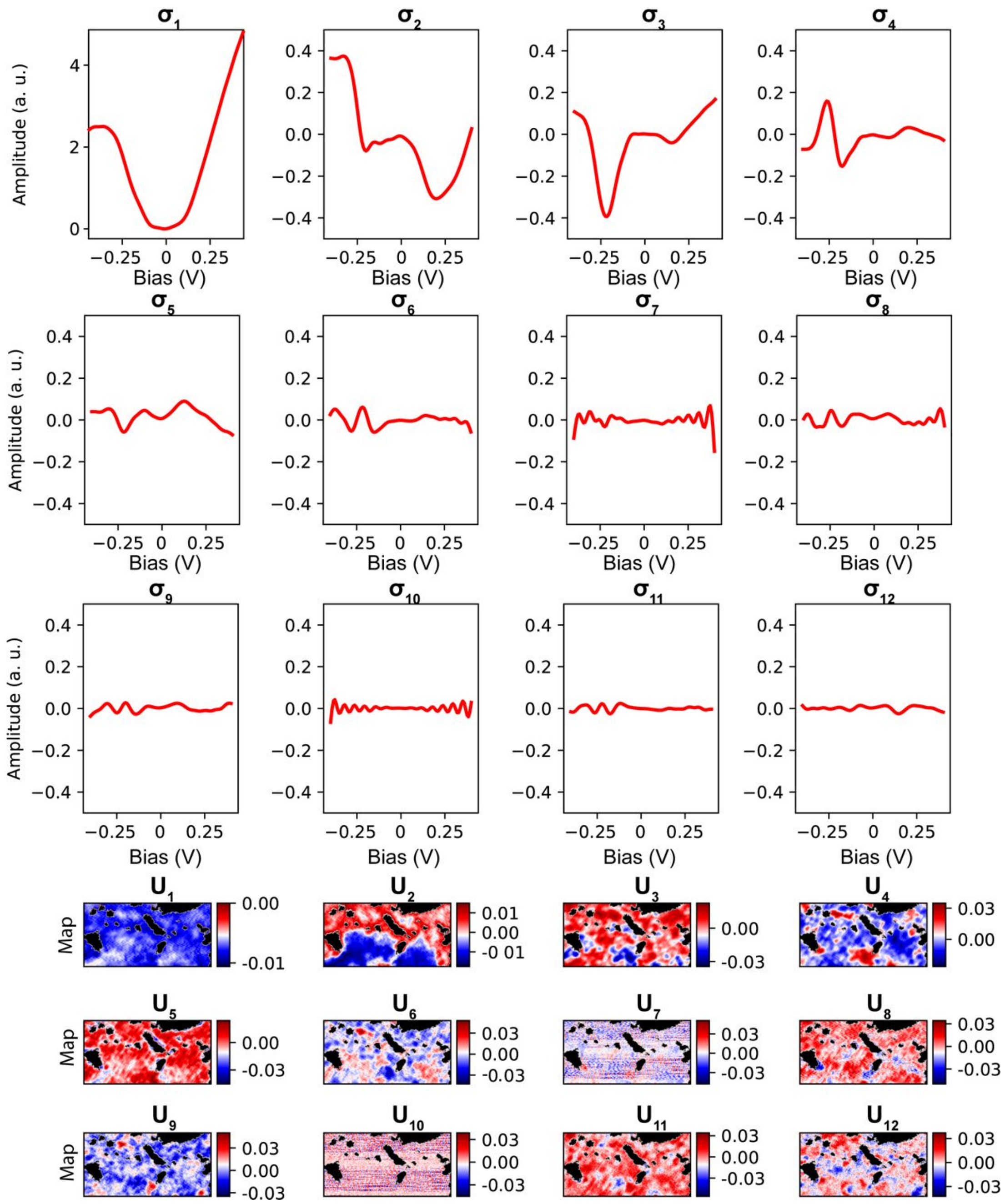

Fig. S6 | Singular value decomposition analysis of the defect-free CITS of single-layer $FeSe_{0.43}S_{0.57}$. The twelve leading of $\sigma_{i=1, 2, \ldots, 12}$ and their corresponding spatial distribution map $U_{i=1, 2, \ldots, 12}$ are obtained by singular value decomposition. As we can see from the amplitude of σ_i, components #1-4 are the most valuable ones.

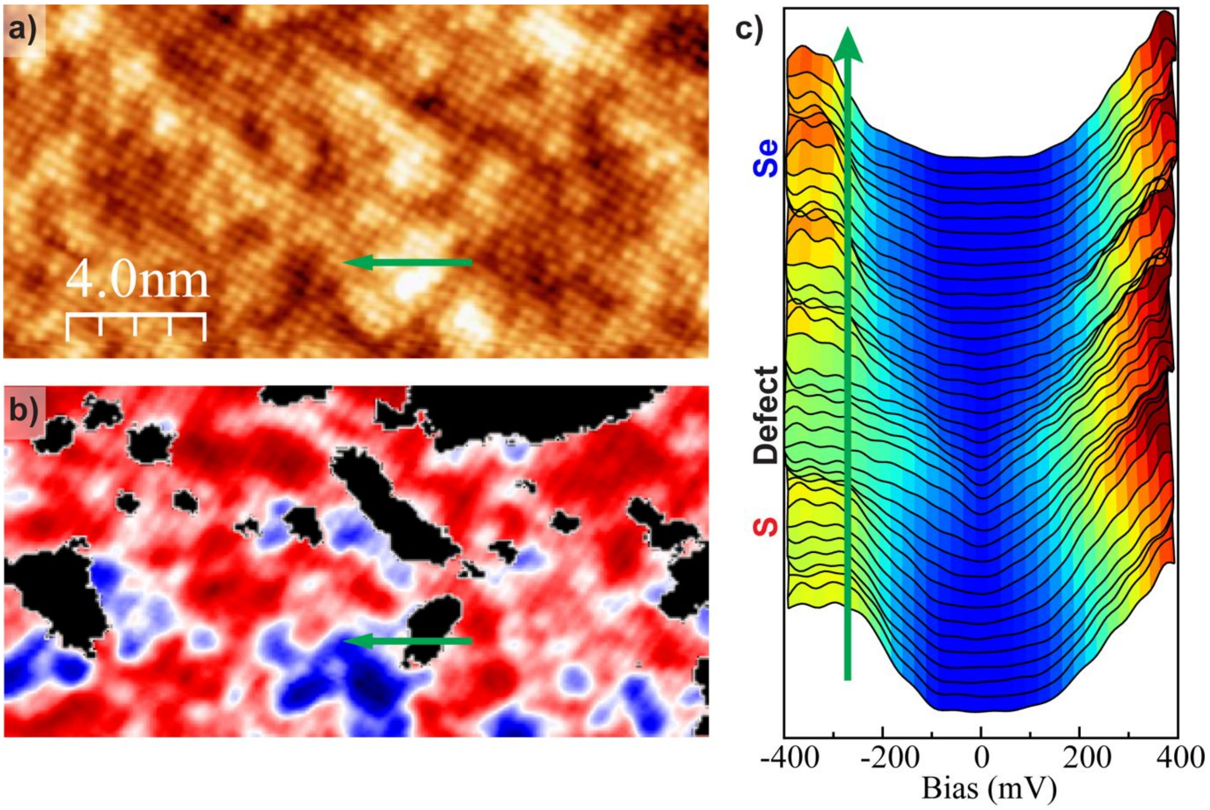

Fig. S7 | Line dI/dV spectra crossing regions of different Se/S ratios in single layer $FeSe_{0.43}S_{0.57}$. (a) Atomic resolution image and (b) spatial distribution map U_3 from the SVD analysis. (c) dI/dV tunneling spectra taken along the green arrow in (a) and (b). Set point: V_{Bias} = 400 meV, I_T = 500 pA.

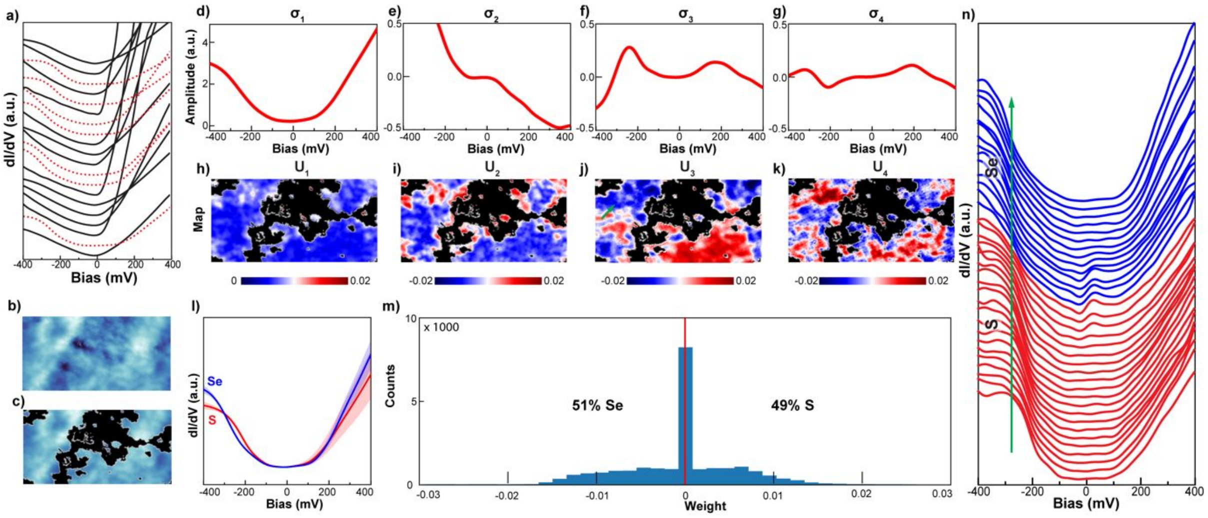

Fig. S8 | Determination of the Se (S) concentration in single-layer $FeSe_{0.51}S_{0.49}$. (a) K-means analysis of CITS spectra. (b) Atomic resolution image and (c) clusters map from K-means plotted over the atomic resolution image. (d) and (h), (e) and (i), (f) and (j), and (g) and (k) are the $\sigma_{i=1, 2, 3, 4}$ and corresponding spatial distribution map $U_{i=1, 2, 3, 4,}$ obtained by singular value decomposition, respectively. (l) The average tunneling spectra with standard deviations at the red and blue locations of map U_3. (m) The statistics of spatial distribution map U_3. (n) dI/dV tunneling spectra taken along the green arrow in (j). Set point: V_{Bias} = 400 meV, I_T = 500 pA.
